# A Matter Of Degrees: Advancing Our Understanding of Acrylamide

**DOI:** 10.1289/ehp.118-a160

**Published:** 2010-04

**Authors:** Angela Spivey

**Affiliations:** **Angela Spivey** writes from North Carolina about science, medicine, and higher education. She has written for *EHP *since 2001 and is a member of the National Association of Science Writers

Until about a decade ago, acrylamide was known only as a constituent of cigarette smoke and of products such as plastics and water treatment chemicals. But in 2002 Swedish scientists were surprised to find this human neurotoxicant and probable carcinogen in many heat-processed foods, especially starchy ones such as potato chips, crackers, and french fries. A flurry of research since then has yielded general advice about reducing formation of acrylamide and other heat-generated food toxicants in home cooking and a few recommendations for healthier eating. Now, in 2010, new acrylamide studies are giving a clearer picture of the extent of exposure to the chemical in the United States. These studies also raise additional questions about whether differences in metabolism make exposure more dangerous in certain populations, including children and people who are obese.

## Revisiting Acrylamide

Acrylamide is one of the hundreds of chemicals known as Maillard reaction products (MRPs), which form when foods are heated at high temperatures. In the Maillard reaction—the chemical process that causes food to brown as it cooks—sugars including glucose, fructose, and lactose react with free amino acids in foods. It’s often MRPs that give food its appetizing colors, smells, and flavors.

Other well-known heat-generated food toxicants include nitrosamines, carcinogens that form in meats and cheeses preserved with nitrites and are increased by frying; heterocyclic amines, carcinogens that form in meat that is well done, fried, or barbecued; and furan and its derivative hydroxymethylfurfural, MRPs that are potential liver toxicants and carcinogens in many foods. A related family of chemicals known as advanced glycation end products (AGEs) are created when acrylamide and other MRPs bind to proteins in food, permanently altering them. AGEs also form in the body, although at lower concentrations.

Acrylamide is known to form adducts to DNA, potentially leading to DNA mutations, so people were understandably alarmed when the compound was found in food. In 2004 the U.S. Food and Drug Administration (FDA) announced an action plan for acrylamide in food that included research on exposures in the U.S. population, more animal studies of the chemical’s carcinogenic and neurotoxic effects, and epidemiologic studies to look for possible associations between acrylamide consumption and cancer. In another initiative, researchers from 14 countries, most of them European, joined together in a research project called HEATOX (Heat-Generated Food Toxicants: Identification, Characterization, and Risk Minimization). This project examined heat-induced food toxicity as a cohesive problem, with a particular emphasis on acrylamide. When it ended in 2007, the project had identified 50 possible heat-generated carcinogens in foods and compiled databases of toxicity probabilities of chemical compounds formed during cooking. Avoiding overcooking was the most important recommendation for home cooks, according to the project’s final leaflet [see p. A163 and A167].

Widespread exposure in the general U.S. population is described in the first large-scale study of acrylamide exposure, published in the February 2010 issue of *EHP* by Hubert Vesper, chief of the Protein Biomarker Laboratory at the Centers for Disease Control and Prevention. Unlike previous studies, this study used a large representative sample—7,166 participants in the 2003–2004 National Health and Nutrition Examination Survey (NHANES). The researchers calculated daily acrylamide exposure based on blood levels of hemoglobin adducts made by acrylamide. The mean exposure levels reported by Vesper and colleagues—0.8 μg/kg/day—are similar to what other researchers have previously reported in smaller studies, he says.

These levels are much lower than the 200 μg/kg body weight found to have no observable neurotoxic effects in humans. However, Vesper’s study suggests more work is needed to determine the factors that affect the conversion of acrylamide to its metabolite glycidamide, especially since several studies indicate glycidamide is responsible for acrylamide’s genotoxic and mutagenic effects. Results from cancer bioassays for acrylamide and glycidamide presented in July 2009 at the annual meeting of the United Kingdom Environmental Mutagen Society also strongly supported a mutagenic role of the latter compound. These assays were conducted by the FDA National Center for Toxicological Research as part of that agency’s acrylamide action plan.

Increased metabolism of acrylamide to glycidamide may be at work in a study published January 2010 in *Biology of Reproduction* in which NIEHS scientist Burhan Ghanayem and colleagues found increased reproductive effects from acrylamide in obese mice compared with lean mice. “When we challenged obese animals versus lean animals with acrylamide, then mated them with normal females, fertility declined at a greater level in obese males,” Ghanayem says. “Also, the dominant male mutation caused by acrylamide was greater and more drastic in pregnancies that resulted from the obese males. Our hypothesis is that more glycidamide formed in the obese animals.”

Ghanayem’s lab is now conducting further studies comparing metabolism of acrylamide in obese versus lean animals. “Although the levels of acrylamide that people consume are lower than those we used in our study, we are concerned that over time this could become cumulative and cause damage,” he says.

## Differences in Acrylamide Metabolization

Another element of acrylamide science that bears closer examination is the question of how children metabolize the chemical compared with adults. It’s been shown previously that children consume a larger amount of food relative to their body mass than adults do. Some researchers (such as Lorelei Mucci of the Harvard School of Public Health and K.M. Wilson, writing in the August 2008 *Journal of Agricultural and Food Chemistry*) have suggested this is particularly true of kid-friendly (and acrylamide-rich) foods such as french fries and potato chips.

In the Vesper study, children aged 3–11 years had higher levels of acrylamide biomarkers than adults, especially older adults. “Children also seem to have a little bit of a different metabolism; they produce more of this toxic glycidamide than older adults,” Vesper says. “We need more research on what this means in terms of health risk, to find out whether the higher levels we see in children are really of importance.”

Another big question yet to be answered is the relationship between intake of acrylamide in food and actual levels of the chemical in the body, Vesper says. In addition to diet, the exposures that can affect levels of acrylamide adducts include cigarette smoking, occupational exposures, and—as Vesper’s *EHP* study showed for the first time—secondhand tobacco smoke. The adduct levels found in his study could have been caused by any of those exposures, not just dietary acrylamide.

Studies that combine measures of adduct levels with food questionnaires may be helpful, Vesper says, but none of the existing questionnaires are designed to specifically assess acrylamide exposure. “Acrylamide in food is highly dependent on the way the food is being prepared, and food preparation is normally not part of food questionnaires. They normally ask questions such as ‘Do you eat french fries?’ but they don’t ask ‘Do you like them very dark?’ for example,” he explains.

Vesper and colleagues reported in the April 2009 *Cancer Causes and Control* there was only a moderate correlation between intake of acrylamide as assessed with a food frequency questionnaire and levels of acrylamide hemoglobin adducts. Vesper is at work with researchers at the International Agency for Research on Cancer on other studies examining the correlation between food intake and biomarkers of acrylamide exposure, as well as studies of differences in acrylamide metabolism associated with polymorphisms of specific genes.

Epidemiologic studies published in 2008 and 2009 have largely found no association between acrylamide exposure and cancers of the colon, rectum, kidney, bladder, or breast. A study published by Henrik Frandsen of Denmark’s National Food Institute and colleagues in the May 2008 *International Journal of Cancer* did show a positive association between higher acrylamide hemoglobin adduct levels and breast cancer risk in postmenopausal women. But, as Mucci and colleagues wrote in an editorial in the 6 May 2009 *Journal of the National Cancer Institute*, the association was statistically significant only among smokers, who are believed to get much more of their acrylamide exposure from smoking than from diet. Previous studies had not found an association between breast cancer and acrylamide exposure as measured by food frequency questionnaires.

## No New Regulations

While research continues, no new laws regarding acrylamide (or any other heat-generated food toxicant) have been forged. “No country has made any regulation on maximum amounts of acrylamide in foods. That may change . . . but I think most countries are trying to avoid that. We will have to wait and see,” says David Lineback, a senior fellow at the Joint Institute for Food Safety and Applied Nutrition, a research and education collaboration between the FDA and the University of Maryland.

Nor have any countries recommended changes to their current guidelines for healthy eating. “Right now we don’t have the data to warrant recommending any changes in dietary habits. We pretty well know that while acrylamide is [neurotoxic], it’s not going to be an issue in foods. We are not going to consume enough to take it into that range,” Lineback says. For now, the FDA’s “best advice for acrylamide and eating” as stated on its Acrylamide Questions and Answers website is simply to follow a healthy diet consistent with the FDA’s *Dietary Guidelines for Americans*. However, for consumers who want to decrease their intake of acrylamide, the FDA also offers an Additional Information on Acrylamide, Diet, and Food Storage and Preparation website with photo guides to illustrate what constitutes “golden yellow” fries and “light brown” toast.

At the state level, California has listed acrylamide as a carcinogen under Proposition 65 since 1990—12 years before it was even discovered in food. After that discovery, food businesses including manufacturers, stores, and restaurants were required to post warnings when they knowingly sell food products that cause exposures to acrylamide. Now the California Office of Environmental Health Hazard Assessment (OEHHA) is proposing to list acrylamide under Prop 65 as a reproductive toxicant as well, on the basis of the 2005 *Monograph on the Potential Human Reproductive and Developmental Effects of Acrylamide* issued by the National Toxicology Program–Center for the Evaluation of Risks to Human Reproduction. The state is accepting public comments on the proposal until 27 April 2010.

Lineback says dietary acrylamide exposure is so widespread—about 30% of the foods we eat contain it—that it’s unlikely we will completely eliminate exposure. “Even if everyone in the United States were to take french fries or potato chips out of their diets, it wouldn’t make a lower overall impact on consumption,” he says. But the food industries in European Union countries and in the United States have researched ways to reduce its formation.

The European countries have shared their data from the start, Lineback says, which has resulted in the “Acrylamide Toolbox,” an informal guidance document developed by the CIAA (Confédération des Industries Agro-Alimentaires de’UE) that details approaches industry can use to reduce acrylamide formation. These include removing some of the sugars in potatoes before frying by blanching them (submerging briefly in boiling water, then cold water) or by soaking potatoes in solutions of glycine, an amino acid that competes with asparagine (the amino acid involved in the reaction that causes acrylamide to form in potato products). Some of these techniques are under further study for their effects on flavor.

## Heating Up: Damage Caused by AGEs

Whereas the main concerns with acrylamide are its carcinogenic and mutagenic effects, the AGES produced by this and other MRPs are under study for their possible contribution to a disease that is rampant today—type 2 diabetes. A large body of literature addresses the health effects of endogenously produced AGEs; it’s well known these compounds accumulate at higher levels in people with diseases such as diabetes and kidney disease, and their presence is associated with aging. But does that mean eating them is bad?

In the past, scientists have debated whether the AGEs we eat in food are simply excreted or whether some of them remain in the body. Today it’s commonly accepted that we absorb enough of the AGEs we eat to modify blood levels, says Helen Vlassara, a professor of geriatrics, medicine, and gene and cell medicine at the Mount Sinai School of Medicine. Vlassara has studied diabetes for more than 30 years but only began examining dietary AGEs a decade ago. But in those 10 years she has become convinced that, to ward off diabetes and kidney disease, you don’t have to avoid eating, say, your 2 eggs every morning. But you must boil or poach them instead of frying them. She believes it’s only a matter of time before it becomes accepted that dietary AGEs increase oxidative stress and inflammation and contribute to disease.

Vlassara reached that conclusion after conducting such work as a study published in the November 2009 *Journal of Clinical Endocrinology and Metabolism* in which she and her colleagues asked a group of healthy people and a group of people with chronic kidney disease about their diets and medical histories. The researchers determined the AGE content of the participants’ diets using a food database they had reported in the August 2004 *Journal of the American Dietetic Association*, in which AGE content was indicated by measuring AGE-*N*-carboxymethyllysine.

The participants who reported eating a diet high in AGEs participated in an interventional study. Half the participants lowered their AGE intake by preparing foods differently at home; this group was instructed to avoid frying, baking, or grilling and to instead boil, poach, stew, or steam their food. The other half prepared their food as usual. Otherwise, the amount and types of food consumed remained the same.

After 4 months, the 15 healthy participants who ate a low-AGE diet showed a significant decrease in serum levels of AGEs and in blood markers of oxidative stress. The 9 participants with kidney disease who participated in the intervention showed similar changes after only 4 weeks.

“We believe that dietary AGEs are a very important environmental factor that is greatly ignored,” says Jaime Uribarri, one of Vlassara’s coauthors. “In healthy subjects, we have measured a whole spectrum of levels of inflammatory and oxidative stress markers. When we modify the diet to reduce formation of AGEs, we change those markers in the right direction.”

Right now Vlassara and Uribarri are conducting a study to determine if they can prevent diabetes in pre-diabetic individuals who eat a low-AGE diet. Uribarri would also like to follow participants for longer periods of time as well as conduct trials in larger groups of people. In addition, he would like to see some of their results replicated in clinical studies by other research groups.

Some researchers say more precise measurement of specific AGEs involved in such clinical studies is needed. John Baynes, professor emeritus of exercise and sport science at the University of South Carolina, points out that in such studies, it’s not certain what factors in the diet are causing inflammation or oxidative stress. “It’s not only AGEs, but also lipoxidation products [formed from cooking of fats] that react with proinflammatory receptors,” he says. “When someone says a diet is high in AGEs, we have to be careful. It probably contains a mixture of products from advanced oxidation chemistry of sugars and lipids. We need some really well-controlled exposures in healthy people that look at biomarkers of inflammation or oxidative stress in response to a high-AGE diet.”

Uribarri agrees, saying, “I’m willing to accept that we should be talking broadly about heat-processed foods. There are hundreds of compounds that we haven’t identified.” He notes that from a practical perspective, however, it doesn’t make any difference whether the problem is caused by AGEs or by lipoxidation products. The clinical guidance would be the same—reduce your intake of foods that are grilled, baked, broiled, or fried—because the same types of foods that are high in AGEs are likely to be high in lipoxidation products as well.

## Possible Mechanisms

Other researchers have begun to show possible mechanisms behind a connection between dietary AGEs and oxidative stress. Jenny Ames of Queen’s University Belfast in Northern Ireland and colleagues reported in the September/October 2009 *Journal of Biochemical and Molecular Toxicology* that by-products of a dairy-related AGE caused an increase in markers of oxidative stress in human cells *in vitro*. The researchers heated cow’s milk protein and glucose to form AGE-casein, then “digested” it using a model system. When they treated human microvascular endothelial cells with the resulting digestion products, they reacted with the RAGE receptor, which is involved in oxidative stress and inflammation, and products of reactive oxygen species increased significantly.

Vlassara has also conducted animal studies to pinpoint specific AGEs that cause problems. In a study published in the March 2010 issue of the *American Journal of Physiology—Cell Physiology*, she and colleagues tested the effect of the AGE methylglyoxal in mice previously fed a low-AGE diet. “We were able to reproduce the effects of a full high-AGE diet by adding only one AGE,” she says. “That increased oxidative stress and caused vascular and kidney problems.”

This study also affected levels of a receptor called AGER1 that Vlassara had previously shown to be active in inhibiting AGEs. “This good receptor was repressed, literally lost, in the animals that ate the diet with methylglyoxal added. But in animals that received the low-AGE diet, the receptor was fully active, and these animals did not succumb to the usual diseases of aging,” she says.

Lineback says food-industry research on AGEs is 20 years behind its research on acrylamide, and that AGEs may be a “storm coming” for the industry. However, short of consuming all foods either raw, boiled, or steamed, “it’s going to be very difficult to talk about the reduction of AGEs in foods,” he says, because AGEs are chemically complex. “Many of these AGEs begin to crosslink proteins, which builds into a much more complex situation. Only a few companies that I know of are monitoring AGEs. On the chemistry side, we don’t know what to do about it because we can’t measure or identify the structures,” he says.

The advancing field of proteomics may provide some progress in that regard. In a review published in the February 2009 issue of the *Journal of Proteome Research*, Baynes and Ames pointed to proteomics as a promising technology for identifying the specific sites at which dietary AGEs modify proteins and determining whether they contribute to pathology. As an exciting start in that direction, Baynes cites a paper published by Fred Regnier of Purdue University in the February 2010 issue of the same journal that identifies the specific sites of modification of some protein adducts caused by AGEs. “It doesn’t provide any evidence that these modifications are functionally significant,” Baynes says, “but it does provide hard data on specific modifications, which is what we need.”

## Figures and Tables

**Figure:**
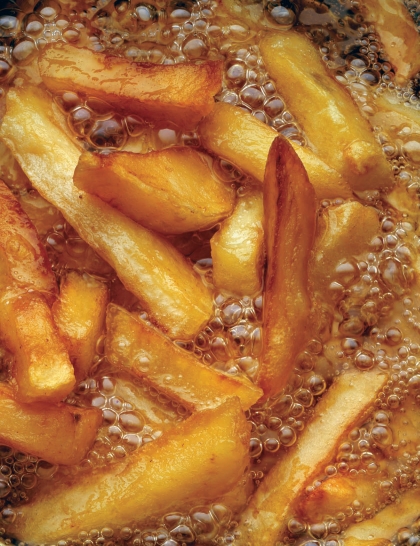


**Figure:**
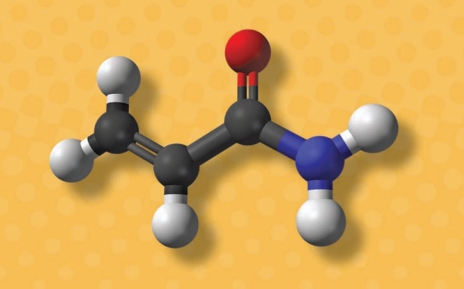
ACRYLAMIDE

**Figure:**
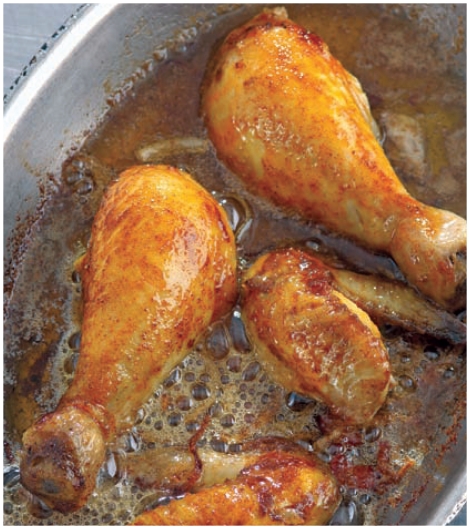
**Although the levels of acrylamide that people consume are lower than those we used in our [rodent] study, we are concerned that over time this could become cumulative and cause damage.** —Burhan Ghanayem National Institute of Environmental Health Sciences

**Figure:**
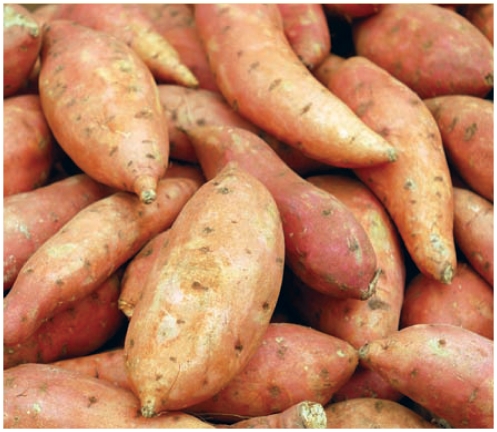
REDUCING HEAT-GENERATED FOOD TOXICANTS One of the end products of the international HEATOX research project was a brochure that detailed some general advice governments could give consumers to reduce formation of acrylamide and other heat-generated food toxicants in home cooking. The U.S. Food and Drug Administration also offers some advice for consumers who want to reduce acrylamide formation in foods. When frying or roasting potatoes, use varieties that are low in sugar.Fry foods in the range of 145–170ºC (293–338ºF).Cook french fries to a golden yellow rather than a golden brown color.Toast bread to the lightest color acceptable.Soak raw potato slices in water for 15–30 minutes before frying or roasting.Do not store raw potatoes in the refrigerator. Don’t let the name fool you—sweet potatoes are relatively low in sugar.

**Figure:**
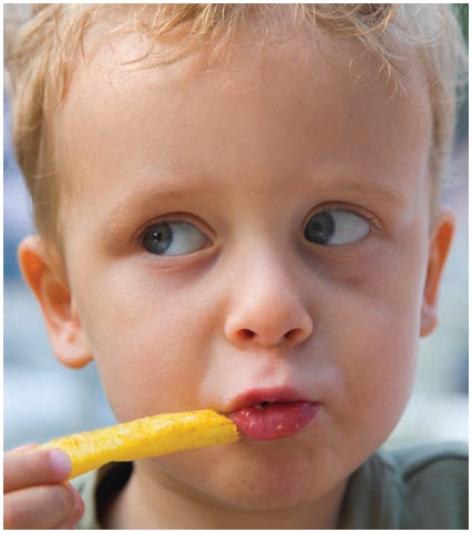
**Acrylamide in food is highly dependent on the way the food is being prepared, and food preparation is normally not part of food questionnaires. They normally ask questions such as “Do you eat french fries?” but they don’t ask “Do you like them very dark?”** —Hubert Vesper Centers for Disease Control and Prevention

**Figure:**
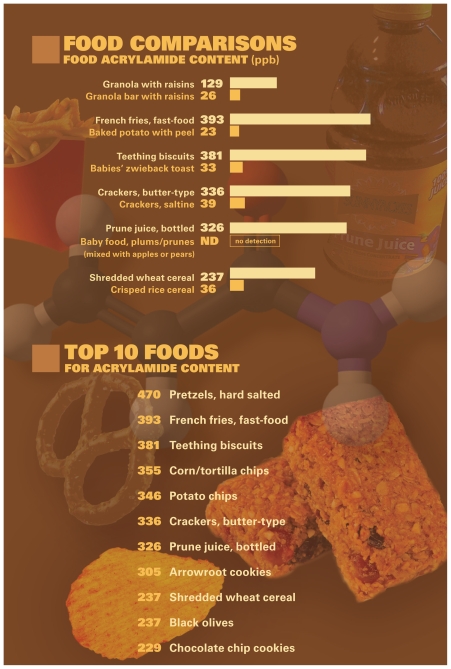
ACRYLAMIDE BY THE NUMBERS Source: FDA. 2006. Survey data on acrylamide in food: Total Diet Study results. Available: http://www.fda.gov/Food/FoodSafety/FoodContaminantsAdulteration/default.htm [accessed 11 March 2010]. These acrylamide levels were measured in the 2006 Total Diet Survey. The Total Diet Survey is an ongoing market basket survey of approximately 280 foods in the U.S. food supply that is conducted by the FDA. Each year the agency collects market baskets from the West, North Central, South, and Northeast regions of the United States. Samples of each food are collected from grocery stores and fast food restaurants in 3 cities within the region, prepared table-ready, and analyzed to produce composite figures for various contaminant and nutrient levels.

**Figure:**
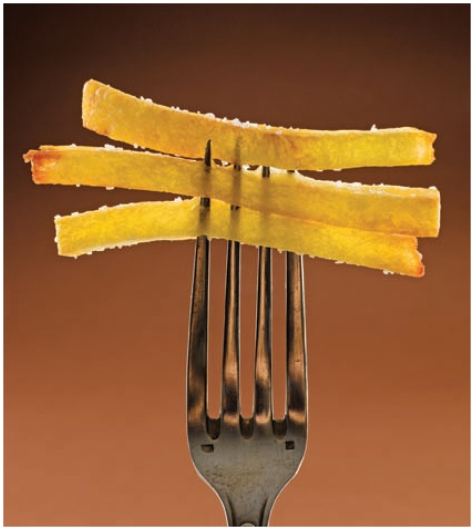
**Right now we don’t have the data to warrant recommending any changes in dietary habits. We pretty well know that while acrylamide is [neurotoxic], it’s not going to be an issue in foods. We are not going to consume enough to take it into that range.** —David Lineback Joint Institute for Food Safety and Applied Nutrition

